# A rare association of serous cystadenoma of the pancreas with mediastinal lipoma: a case report

**DOI:** 10.4076/1757-1626-2-7165

**Published:** 2009-08-11

**Authors:** Sankar Subramanian, Subramanian Marappagounder, Deepu Rajkamal Selvaraj, Babu Elangovan

**Affiliations:** Department of Surgical Gastroenterology, Sri Ramachandra Medical College & Research Institute, Sri Ramachandra UniversityPorur, Chennai, 600116, Tamil NaduIndia

## Abstract

Cystic lesions of pancreas include a myriad of different conditions ranging from the common pseudocyst to unusual cystic neoplasm. With the development of better imaging modalities, cystic neoplasms are diagnosed with greater frequency and accuracy leading on to better understanding of the natural course of these lesions. Serous cystadenoma is one of the rare neoplasms of the pancreas that is unique for its benign nature. Most of the time surgery is indicated for the symptom of pain when the lesion enlarges. Surgery may also be indicated due to the fact that, it may not always be possible to dogmatically differentiate it from the potentially malignant counterpart radiologically. One of the interesting aspects about serous cystadenoma is its association with other systemic disorders like von Hippel Lindau syndrome. Herein we report a rare association of serous cystadenoma with mediastinal lipoma, which has not been reported in the literature.

## Case presentation

**Figure 1.**
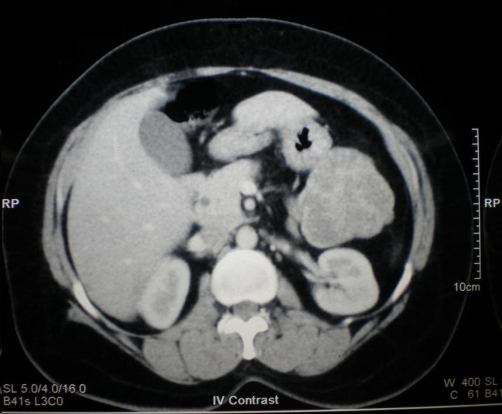
Well-defined lesion arising from the tail of pancreas predominantly hypodense with heterogenous contrast enhancement.

**Figure 2.**
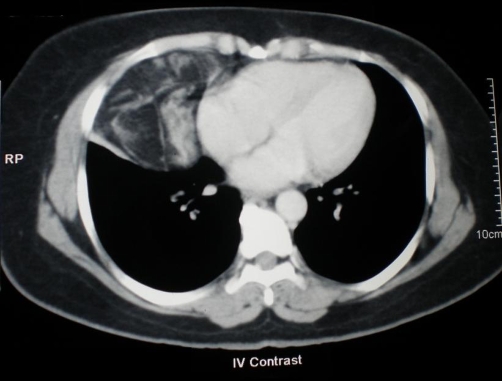
Lesion in the anterior mediastinum which had low CT attenuation values suggestive of a lipoma.

**Figure 3.**
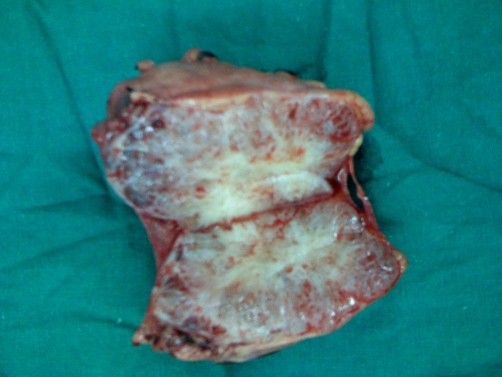
Resected lesion showed a central scar with honeycomb appearance.

A 50-year-old South Indian female presented with vague abdominal pain of six months duration. Clinical examination revealed a vague mass in the left upper quadrant. There were no palpable mass in the neck or chest. Blood investigations were found to be normal. Ultrasound abdomen showed a mixed echogenic lesion in the tail of the pancreas measuring 7 × 5 cm. CECT (Contrast enhanced computerized tomogram) showed a well defined lesion arising from the tail of the pancreas predominantly hypodense with heterogenous contrast enhancement (Figure 1). CECT also showed a heterogenous lesion in the anterior mediastinum which had predominantly low attenuation value in the region of -60 to -100 suggestive of lipoma (Figure 2). Patient underwent distal pancreatectomy and the postoperative period was uneventful. The gross appearance of the cut section of the resected lesion showed a central scar with honeycomb appearance (Figure 3). Histopathologically the tumor was confirmed as serous cystadenoma. Patient had an uneventful postoperative period and doing fine in the follow up. As the mediastinal lesion was asymptomatic and benign it was not operated. The association of serous cystadenoma and mediastinal lipoma has not been reported in the literature. This patient was not exhibiting any other features of von Hippel Lindau syndrome.

## Discussion

Cystic lesions of the pancreas constitute a wide variety of conditions which include pseudocysts, true congenital cysts and cystic neoplasms [1]. Cystic neoplasm accounts for 10% of the cystic lesions of the pancreas [2]. Diagnosis of these lesions has become more frequent and accurate due to advancement in imaging technology. Mucinous cystadenoma, serous cystadenoma, intraductal mucinous neoplasm and Frantz tumor are some of the types of cystic neoplasm of the pancreas. The natural course and malignant potential of these different neoplasms are different.

Serous cystadenoma of the pancreas, also called microcystic adenoma or glycogen rich adenoma is unique for its benign nature with little malignant potential [3]. Pancreatic serous cystadenoma are classically rare. Over a 55-year period in a large population examined at the Mayo Clinic, only 40 patients underwent surgical treatment for this disease [4]. Serous cystadenoma that are symptomatic are usually large in size and the most common symptom is pain. Serous cystadenoma discovered incidentally during imaging or intra-operatively are small in size. Male-female-prevalence ratio of serous cystadenoma is 1:2 to 1:3. The mean patient age at presentation is 62 years (range, 35-84 years) [3]. Serous cystadenomas occur most commonly in the pancreatic head and body and rarely involve the main pancreatic duct. They are lined by a uniform layer of glycogen-secreting cuboidal cells. A central stellate scar with calcification and a grapelike cluster of cysts(honey comb appearance) that are less than 5mm and external lobulation strongly suggests serous cystadenoma. However, the imaging features of these entities can overlap considerably. Therefore, an analysis of the cytology and cystic fluid is often required for diagnosis. In fact, approximately 10% of all serous cystadenomas have cystic components larger than 2 cm and cannot be distinguished from mucinous cystic neoplasms.

On ultrasound the lesion appears as a solid echogenic mass due to the myriad of interfaces produced by the numerous cysts. CECT shows a swiss cheese appearance with gentle external lobulation. A central stellate scar with calcification may be seen in 20% of the cases. The lesion appears hypointense on T1 weighted sequence and hyperintense on T2 weighted sequence of MRI.

Serous cystadenoma may be associated with other disorders which include von Hippel Lindau [5], Evans syndrome [6], intestinal hemangiomas [7] and other entities [8]. The case reported here is rare for the association of serous cystadenoma with mediastinal lipoma which has not been reported in the literature.

## Abbreviation

CECT: contrast enhanced computerized tomogram

## References

[bib-001] Howard JM (1989). Cystic neoplasm and true cysts of pancreas. Surg Clin North Am.

[bib-002] Warshaw AC, Fernandez del Castillo C, Rattner DW, Zinner MA, Ashley SW (2001). Pancreatic cysts, pseudocysts, fistulas. Maingot’s abdominal operations.

[bib-003] Compagno J, Oertel JE (1978). Microcystic adenoma of the pancreas (glycogen-rich cystadenoma): a clinicopathologic study of 34 cases. Am J Clin Pathol.

[bib-004] Pyke CM, van Heerdan JA, Colby TV, Sarr MG, Weaver AL (1992). The spectrum of serous cystadenoma of the pancreas. Ann Surg.

[bib-005] Hortan WA, Wong V, Eldridge R (1976). Von Hippel-Lindau disease: clinical pathological manifestation in nine families with 50 affected members. Arch Intern Med.

[bib-006] Doll DC, List AF, Yarbro JW (1987). Evan’s syndrome associated with microcystic adenoma of the pancreas. Cancer.

[bib-007] Rosenbaum H, Conolly PJ, Climie ARW, Reveno WS (1963). Pancreatic cystadenoma with intestinal hemorrhage. Am J Roentgenol.

[bib-008] Soloway HB (1965). Constitutional abnormalities associated with pancreatic cystadenoma. Cancer.

